# An Audit and Feedback Intervention for Reducing Antibiotic Prescribing in General Dental Practice: The RAPiD Cluster Randomised Controlled Trial

**DOI:** 10.1371/journal.pmed.1002115

**Published:** 2016-08-30

**Authors:** Paula Elouafkaoui, Linda Young, Rumana Newlands, Eilidh M. Duncan, Andrew Elders, Jan E. Clarkson, Craig R. Ramsay, Translation Research in a Dental Setting (TRiaDS) Research Methodology Group

**Affiliations:** 1 NHS Education for Scotland (NES), Dundee Dental Education Centre, Frankland Building, Dundee, United Kingdom; 2 Dental Health Services Research Unit (DHSRU), University of Dundee, Park Place, Dundee, United Kingdom; 3 Health Services Research Unit (HSRU), University of Aberdeen, Health Sciences Building, Foresterhill, Aberdeen, United Kingdom; 4 NMAHP Research Unit, Glasgow Caledonian University, Cowcaddens Road, Glasgow, United Kingdom; University of Geneva Hospitals and Medical School, SWITZERLAND

## Abstract

**Background:**

Dentists prescribe approximately 10% of antibiotics dispensed in UK community pharmacies. Despite clear clinical guidance, dentists often prescribe antibiotics inappropriately. This cluster-randomised controlled trial used routinely collected National Health Service (NHS) dental prescribing and treatment claim data to compare the impact of individualised audit and feedback (A&F) interventions on dentists’ antibiotic prescribing rates.

**Methods and Findings:**

All 795 antibiotic prescribing NHS general dental practices in Scotland were included. Practices were randomised to the control (practices = 163; dentists = 567) or A&F intervention group (practices = 632; dentists = 1,999). A&F intervention practices were allocated to one of two A&F groups: (1) individualised graphical A&F comprising a line graph plotting an individual dentist’s monthly antibiotic prescribing rate (practices = 316; dentists = 1,001); or (2) individualised graphical A&F plus a written behaviour change message synthesising and reiterating national guidance recommendations for dental antibiotic prescribing (practices = 316; dentists = 998). Intervention practices were also simultaneously randomised to receive A&F: (i) with or without a health board comparator comprising the addition of a line to the graphical A&F plotting the monthly antibiotic prescribing rate of all dentists in the health board; and (ii) delivered at 0 and 6 mo or at 0, 6, and 9 mo, giving a total of eight intervention groups. The primary outcome, measured by the trial statistician who was blinded to allocation, was the total number of antibiotic items dispensed per 100 NHS treatment claims over the 12 mo post-delivery of the baseline A&F. Primary outcome data was available for 152 control practices (dentists = 438) and 609 intervention practices (dentists = 1,550). At baseline, the number of antibiotic items prescribed per 100 NHS treatment claims was 8.3 in the control group and 8.5 in the intervention group. At follow-up, antibiotic prescribing had decreased by 0.4 antibiotic items per 100 NHS treatment claims in control practices and by 1.0 in intervention practices. This represents a significant reduction (-5.7%; 95% CI -10.2% to -1.1%; *p* = 0.01) in dentists' prescribing rate in the intervention group relative to the control group. Intervention subgroup analyses found a 6.1% reduction in the antibiotic prescribing rate of dentists who had received the written behaviour change message relative to dentists who had not (95% CI -10.4% to -1.9%; *p* = 0.01). There was no significant between-group difference in the prescribing rate of dentists who received a health board comparator relative to those who did not (-4.3%; 95% CI -8.6% to 0.1%; *p* = 0.06), nor between dentists who received A&F at 0 and 6 mo relative to those who received A&F at 0, 6, and 9 mo (0.02%; 95% CI -4.2% to 4.2%; *p* = 0.99). The key limitations relate to the use of routinely collected datasets which did not allow evaluation of any effects on inappropriate prescribing.

**Conclusions:**

A&F derived from routinely collected datasets led to a significant reduction in the antibiotic prescribing rate of dentists.

**Trial Registration:**

Current Controlled Trials ISRCTN49204710

## Abstract 2

In this cluster randomized controlled trial, Linda Young and colleagues investigate the impact of an audit and feedback intervention for reducing antibiotic prescribing by dentists.

## Author Summary

**Why Was This Study Done?:**

- Antimicrobial resistance is an increasingly serious threat to global public health and patient safety.
- Dentists prescribe around 10% of antibiotics dispensed in UK community pharmacies, but, despite clear clinical guidance, dentists often prescribe antibiotics inappropriately in the absence of clinical need.
- This study evaluated the impact of using routine data to audit and provide dentists with individualised feedback comprising a line graph plotting their monthly antibiotic prescribing rate on dentists’ antibiotic prescribing.

**What Did the Researchers Do and Find?:**

- All 795 antibiotic prescribing NHS general dental practices in Scotland were randomly allocated to receive or not to receive individualised feedback.
- At follow-up, the antibiotic prescribing rate of dentists who received individualised feedback was 5.7% lower than the antibiotic prescribing rate of dentists who did not receive individualised feedback.
- Feedback that included a written message synthesising and reiterating national guidance recommendations had the greatest effect.

**What Do These Findings Mean?:**

- Individualised graphical feedback derived from routinely collected data can significantly reduce the antibiotic prescribing rate of dentists.
- A key limitation of using routine data is that it was not possible to evaluate the impact on the quality or appropriateness of dentists’ antibiotic prescribing.
- The feedback provided in this study is a relatively straightforward, low-cost public health and patient safety intervention that could potentially help the entire healthcare profession address the increasing challenge of antimicrobial resistance.

## Introduction

Antimicrobial resistance is an increasingly serious threat to global public health and patient safety, resulting in increased morbidity, mortality, and health care costs [1,2]. Increased use of antibiotics in medicine, dentistry, and agriculture is a major contributor to the spread of antimicrobial resistance [3,4]. Dentists are responsible for approximately 10% of all antibiotics dispensed in UK community pharmacies [5–8]. Despite clear clinical guidance [9,10], evidence demonstrates that dentists often prescribe antibiotics inappropriately in the absence of clinical need [5,7,11,12].

There is an urgent need to change health professionals’ prescribing behaviour, but the effectiveness of strategies to change the behaviour of health professionals is variable. A systematic review [13] that included 140 randomised controlled trials showed that audit and feedback (A&F) has small to moderate effects on health professionals’ behaviour. However, the evidence informing its effectiveness for changing antibiotic prescribing behaviour within the primary-care setting was sparse, with only four of the trials studying the effect of feedback on prescribing within primary care. The review authors concluded that A&F could lead to small but important improvements and future studies should directly compare the effectiveness of different ways of providing feedback.

National guidance [9] to improve primary care dental prescribing was first published and distributed to all dentists in Scotland by the Scottish Dental Clinical Effectiveness Programme (SDCEP) in April 2008. The guidance states that dental antibiotic prescribing must be kept to a minimum and recommends that the first step in the treatment of bacterial infections should be the use of local measures (e.g., drain pus in a dental abscess). Antibiotics are only indicated if there are signs of spreading infection or systemic involvement and should be used in conjunction with local measures.

Recognising the continued knowledge-to-practice gap and to support the implementation of SDCEP recommendations for antibiotic prescribing, the Translation Research in a Dental Setting (TRiaDS) programme [14], which develops and evaluates interventions for improving dental healthcare in Scotland, identified antibiotic prescribing by dentists as a priority area for research. The RAPiD (Reducing Antibiotic Prescribing in Dentistry) trial reported here is, to our knowledge, the first dental primary care study to randomise all practices in a country using national NHS dental prescribing and treatment claim data to compare the effectiveness of individualised A&F interventions for the translation into practice of national guidance recommendations on antibiotic prescribing. In addition, a secondary objective was to explore dentists’ experiences of and responses to the individualised A&F interventions and to facilitate understanding of the processes associated with antibiotic prescribing in dentistry.

## Methods

### Ethical Approval

The East of Scotland Research Ethics Service reviewed the study and, in line with UK Governance Arrangements for Research Ethics Committees (GAfREC), confirmed ethical review or approval by an NHS REC was not required (Ref: 11/GA/229). In addition, the protocol was submitted to the NHS Research Scotland Permissions Coordinating Centre and reviewed by the Tayside Medical Science Centre Research and Development (R&D) office. They classified the RAPiD trial as service development/audit and confirmed that it did not require R&D registration, formal review, or approval. Confirmation was received from all 14 Scottish Health Boards that they had been notified of the study and had added it to their Clinical Governance and Quality Improvement records. The data used to derive the graphical A&F contained no identifiable information about the patients who were prescribed an antibiotic or about the patients who received treatment under a participating dentist’s list number, and, therefore, informed consent from patients was not required.

### Trial Registration

The study was registered with Current Controlled Trials ISRCTN49204710, available at www.conrolled-trials.com and a detailed protocol published (RAPiD Trial Protocol) prior to the final data collection and before any outcomes were analysed.

### Trial Design

Details of the trial design and methods have been described in detail elsewhere [15]. To summarise, RAPiD was a 12-mo partial factorial cluster randomised controlled trial conducted in NHS General Dental Practices across Scotland.

Practices were randomly allocated to the control group (no A&F) or to the intervention group. Intervention group practices were evenly allocated to one of two A&F groups (individualised graphical A&F with or without a written behaviour change message). In each of these two groups, practices were simultaneously allocated to receive A&F: (i) with or without a health board comparator; and (ii) at 0 and 6 mo or at 0, 6, and 9 mo, giving a total of 8 intervention groups. In each intervention practice, all dentists received their own individualised graphical A&F according to their practice’s allocation.

**Fig 1:**
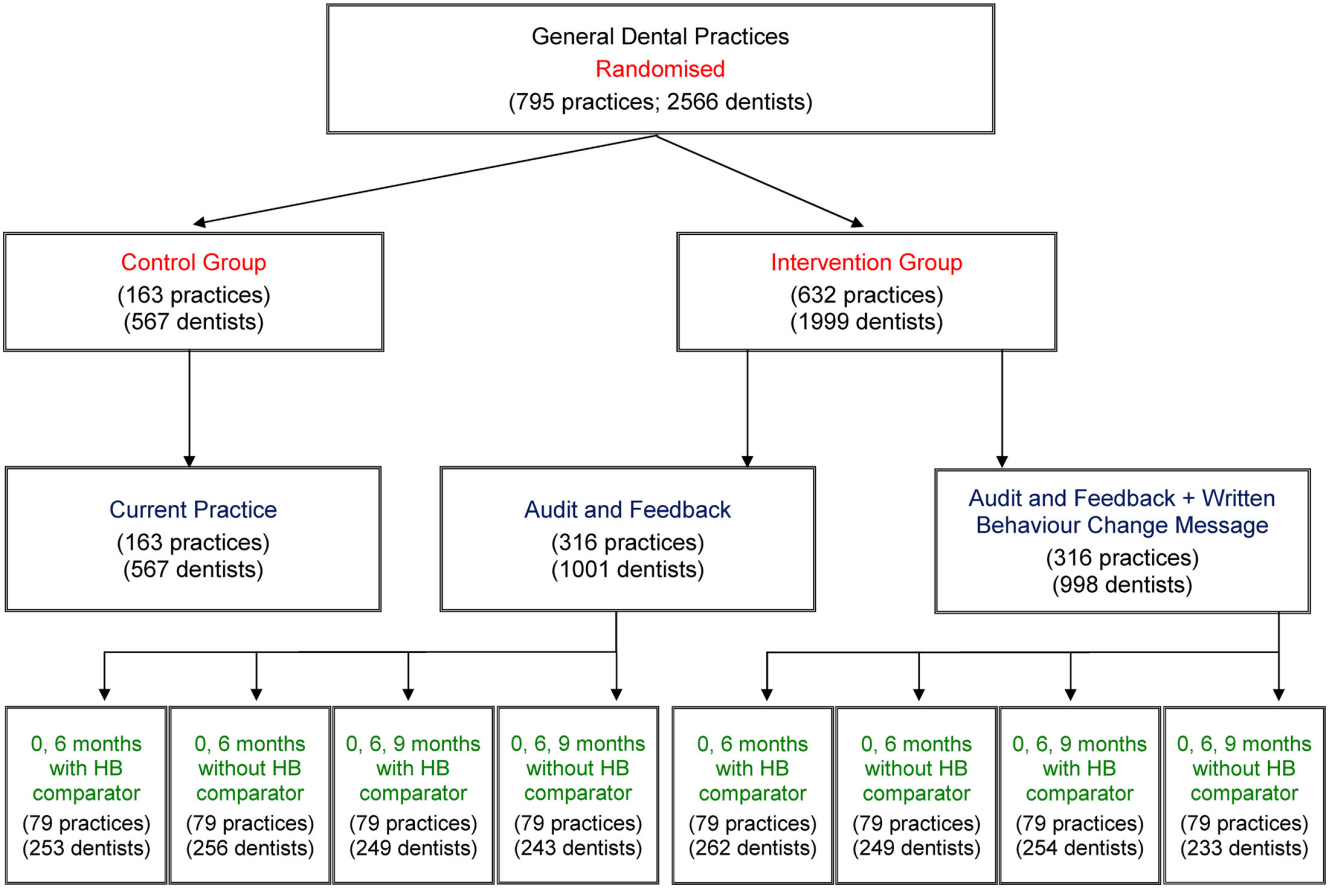
RAPiD trial design. Description of interventions: Audit and feedback—a line graph plotting an individual dentist’s monthly antibiotic prescribing rate. Written behaviour change message—text added below the individualised line graph synthesising and reiterating national guidance recommendations for antibiotic prescribing. HB comparator—addition of a line to the individualised line graph plotting the monthly antibiotic prescribing rate of all dentists in that dentist’s health board. 0, 6 months—allocated intervention delivered at months 0 and 6. 0, 6, 9 months—allocated intervention delivered at months 0, 6, and 9.

A cluster design was used to reduce contamination within dental practices. A factorial design was used to assess the effect of three A&F interventions (i.e., inclusion of a written behaviour change message, inclusion of a health board comparator, and varying the interval between receiving A&F). A control group was included to test the effectiveness of any form of A&F strategy. The trial design is depicted in Fig 1.

### Study Population and Eligibility

All NHS General Dental Practices across the 14 health boards in Scotland were assessed for eligibility to be included in the study. Practice addresses and the names of all individual dentists working in these practices were identified from the Management Information and Dental Accounting System (MIDAS) database. MIDAS contains contact information for all primary care dental practices and dentists in Scotland and details all payment claims made by dentists for the NHS treatment provided to their patients.

A minimum of 6 mo of NHS treatment claim data in the 12 mo prior to the delivery of the baseline intervention at month 0 was required for practices to be included in the trial. Practices in mainland health boards with NHS salaried dentists were excluded. On the Scottish mainland, NHS salaried dentists are generally employed by their health board to provide community and emergency dental services. The range of treatments provided in these services is more limited than in the General Dental Service, and, therefore, salaried dentists were used as a proxy to identify these types of practices. Predominantly due to geography, the majority of dental services in the island health boards are provided by NHS salaried dentists. However, in contrast to salaried dentists in mainland health boards, salaried dentists in island health boards also provide general dental services. Therefore, all practices in the island health boards were included. Within each included practice, all dentists listed in MIDAS in the month prior to the intervention being delivered at month 0 were included in the trial.

### Intervention Development

#### Graphical individualised audit and feedback

The graphical A&F for an individual dentist comprised a line graph plotting the individual dentist’s monthly antibiotic prescribing rate presented on a single side of paper.

The graph was derived using two routinely collected electronic healthcare datasets held centrally by the Information Services Division of NHS National Services Scotland.

1. The Prescribing Information System for Scotland (PRISMS) database. PRISMS contains information for all primary care prescription items dispensed in community pharmacies since April 2004. For each dentist, their monthly antibiotic prescribing volume was the number of antibiotic items (British National Formulary, Section 5.1 [16]) prescribed and dispensed in a community pharmacy each month. Each antibiotic item represents a single prescription item for a course of antibiotics.
2. The MIDAS database contains information relating to all NHS treatment claims made by dentists in the General Dental Service since 1990. Each NHS treatment claim represents a claim for payment for the provision of single course of NHS dental treatment to an individual patient. The course of treatment provided is determined by the patient’s oral health needs and may be delivered at one or more appointments dependent on the number of individual treatments required (e.g., examination only, examination plus a filling, etc.).

A list number is allocated to all primary care dentists in Scotland by their health board, and all patients attending a dentist are registered under that dentist’s list number. List numbers are used as the dentist identifier in both the PRISMS and MIDAS databases and were used as the single common identifier to link each dentist’s prescribing data from PRISMS to their NHS treatment claim data from MIDAS. For each individual dentist, the prescribing rate was calculated as the monthly number of antibiotic items recorded in PRISMS divided by the mean monthly number of NHS treatment claims recorded in the MIDAS database (multiplied by 100).

#### Written behaviour change message

The written behaviour change message synthesised and reiterated national guidance recommendations for dental antibiotic prescribing. This message was placed immediately below the A&F line graph on the same side of the paper.

To construct this message, SDCEP guidance recommendations for antibiotic prescribing when managing patients with bacterial infections were coded for the presence/absence of behaviour change techniques (BCTs), using the 2012 BCT taxonomy [17,18], by a researcher trained in its use. Two BCTs were identified and selected for inclusion in the behaviour change message: (1) instruction on how to perform the behaviour; and (2) provide information about health consequences of performing the behaviour. When possible, the exact wording from the SDCEP prescribing guidance was used. Full details of the wording and the development can be found in S1 Text. An example A&F chart including the written behaviour change message is provided in S1 Fig.

#### Health board comparator

The health board comparator was the inclusion of an additional line to the A&F graph plotting the monthly antibiotic prescribing rate of all dentists in an individual dentist’s health board. Monthly prescribing rates for health boards were calculated using PRISMS prescribing data and MIDAS NHS treatment claim data in the same way as for each individual dentist but were based on the total number of antibiotic items dispensed and total number of NHS treatment claims within each health board.

#### The interval between receiving feedback

The interval between receiving A&F was varied according to allocation (Fig 1), with A&F received at either 0 and 6 mo or at 0, 6 and 9 mo.

### Intervention Delivery

MIDAS and PRISMS data are updated routinely on a monthly basis with a time lag of between 2 and 3 mo. Updates of data were received by the trial office every month throughout the duration of the trial, and the A&F delivered to dentists at 0 and 6 mo or at 0, 6, and 9 mo contained the most recently available data. All A&F was delivered by post to the dentist’s practice address.

The initial A&F delivered to dentists in intervention practices at month 0 (01 May 2013) contained retrospective prescribing rates taken from the most recently available previous 14 mo (01 November 2011 to 31 December 2012). The A&F delivered at month 6 (01 November 2013) contained 20 mo of data (01 November 2011 to 30 June 2013). The A&F delivered at month 9 (01 February 2014) contained 23 mo of data (01 November 2011 to 30 September 2013).

### Outcomes

The linked prescribing/NHS treatment claim dataset was used to determine the outcomes for each dentist within a dental practice. The primary outcome, measured by the trial statistician, who was blinded to allocation, was the total number of antibiotic items (from Section 5.1 of the British National Formulary [16]) dispensed per 100 NHS treatment claims over the 12 mo from May 2013 to April 2014.

The SDCEP guidance [9] advises against the use of antibiotics as prophylaxis for the prevention of infective endocarditis or to prevent infection in patients with prosthetic joints. The most common antibiotic used by dentists as prophylaxis in these circumstances is amoxicillin 3g. The guidance also advises against the routine use of broad spectrum antibiotics in dentistry. Therefore, for each dentist, the secondary outcomes were the total number of amoxicillin 3g dispensed per 100 NHS treatment claims over the 12 months from May 2013 to April 2014 and the total number of broad spectrum antibiotics (clindamycin, co-amoxiclav, clarithromycin, cefalexin, and cefradine) dispensed per 100 NHS treatment claims over the 12 months from May 2013 to April 2014.

Because measurement of an antibiotic item provides no information about the dosage contained within each item, the defined daily dose (DDD) prescribing rates over the same period were also calculated for the primary outcome and the additional secondary outcomes. The DDD is a statistical measure of drug consumption defined by the World Health Organisation Collaborating Centre for Drug Statistics Methodology as “the assumed average maintenance dose per day for a drug used for its main indication in adults” [19]. It facilitates evaluation of trends in drug consumption and comparisons of consumption across different populations.

### Sample Size

The required sample size to achieve 80% power (with two-sided alpha of 2.5% allowing for multiple comparisons) to detect a 10% difference in overall antibiotic prescribing between intervention groups was 316 per group. This applied to the comparison between A&F only and A&F with an additional written behaviour change message, the comparison between those with and without a health board comparator, and the comparison between A&F at 0, 6, and 9 mo versus 0 and 6 mo only. Therefore, 632 practices were required to receive an A&F intervention with 79 practices in each of the eight sub-level experimental units. There were 795 practices eligible to be included in the trial, which left 163 practices in the control arm. The comparison between the control group (*n* = 163) and the intervention group (*n* = 632) had 80% power to detect a 12% decrease in overall antibiotic prescribing. The study was not powered to detect realistic two-way interaction effects between behavioural components.

The sample size and power calculations were based on aggregated practice level antibiotic prescribing activity recorded in PRISMS for the 1,799 dentist list numbers in Scotland known to be prescribing throughout the year ending June 2010. The mean number of antibiotic items prescribed per list was 141.1 with a standard deviation of 140.9. The correlation with the year ending June 2009 was 0.91, and the calculations were adjusted using the correction described by Borm et al. for trials with correlated data [20].

### Randomisation and Allocation

The unit of randomisation was the dental practice. All eligible dental practices (*n* = 795) were simultaneously randomised by the trial statistician at the beginning of the trial, prior to any baseline feedback being distributed. The statistician was blinded to the identity of the practices. The allocation schedule for random assignment was computer generated. Practices were ordered randomly, with the first 632 practices being allocated to an A&F intervention and the remaining practices being allocated to the control group (*n* = 163). Each of the 632 intervention practices was allocated to one of eight subgroups with an even allocation so that 79 practices were randomised to each subgroup (Fig 1). Randomisation was stratified by single-handed/multi-handed practices. Single-handed practices are practices in which there is only one dentist, whereas in multi-handed practices there is more than one dentist.

### Statistical Analysis

All statistical analyses were conducted in Stata 13. A single principle analysis was conducted using 12 mo post baseline intervention data (i.e., covering up to 30 April 2014). The analysis estimated the effect of the overall A&F intervention compared with current practice and also estimated the differential effect of separate elements of the intervention (i.e., inclusion of the TRiaDS written behaviour change message, inclusion of a health board comparator, and frequency of feedback) within the intervention group. Main effects analyses of covariance on the 12-month prescribing rate were performed, adjusting for the pre-intervention annual prescribing rate and practice size (defined as single-handed/multi-handed). Two-way interaction terms were estimated when comparing the differential effect of separate elements of the intervention. All outcomes were weighted by the number of NHS treatment claims submitted by the dentist during the year from May 2013 to April 2014, and the analyses were clustered by dental practice. Effect sizes were calculated as the difference in the prescribing rate between groups and also the equivalent percentage reduction from baseline. The same approach was used for all prescribing behaviours under investigation. All analyses adjusted for clustering of dentists within practice using the Huber-White robust standard error procedure in Stata 13 [21]. Model residuals were checked to assess goodness of model fit.

Additionally, for the primary outcome, we investigated whether larger effects of the interventions were observed for dentists reporting higher pre-intervention levels of prescribing (when the annual prescribing rate was above the upper quartile). The intra-cluster correlation coefficient to measure relatedness of the data within dental practices was also determined, and to test for a reduction in the spread of prescribing levels, Levene's test for equality of variances between pre-intervention and intervention phases was applied.

### Process Evaluation

Full details of the process evaluation methods have been reported elsewhere [15]. To summarise, 30 semi-structured audio-recorded telephone interviews from a purposive sample of dentists working within eligible dental practices (i.e., practices allocated to an A&F intervention group or allocated to the control group) were conducted. Potential participants were identified from the linked MIDAS and PRISMS data. Three hundred potential participants (100 low prescribers, 100 medium, and 100 high) were sampled using implicit stratification, ensuring representativeness based on the following baseline factors: health board, practice prescribing profile (e.g., all dentists in the practice are high/medium/low prescribers or a mixture of high/medium/low prescribers), and practice size (i.e., single-handed/multi-handed). Throughout recruitment, diversity variables were tracked in order to inform ongoing sampling of potential participants to maximise representativeness across these factors. From the 64 dentists contacted to take part, 30 agreed to be interviewed (47% response rate).

The interview process was ceased after the 30th interview, when no new information was obtained. The transcribed interviews were content-analysed by a single researcher using the qualitative data analysis software package NVivo 10. The Consolidated Framework for Implementation Research [22] and the Theoretical Domains Framework [23,24] for health psychology were used as coding frameworks. A second researcher also coded 10% of the transcripts independently to ensure fidelity of the coding system. All the coding was discussed and agreed on by three researchers. This paper presents the findings of dentists’ experiences of and responses to the A&F interventions. The findings on the processes associated with antibiotic prescribing are published separately [25].

## Results

### Participants and Intervention Delivered

**Fig 2:**
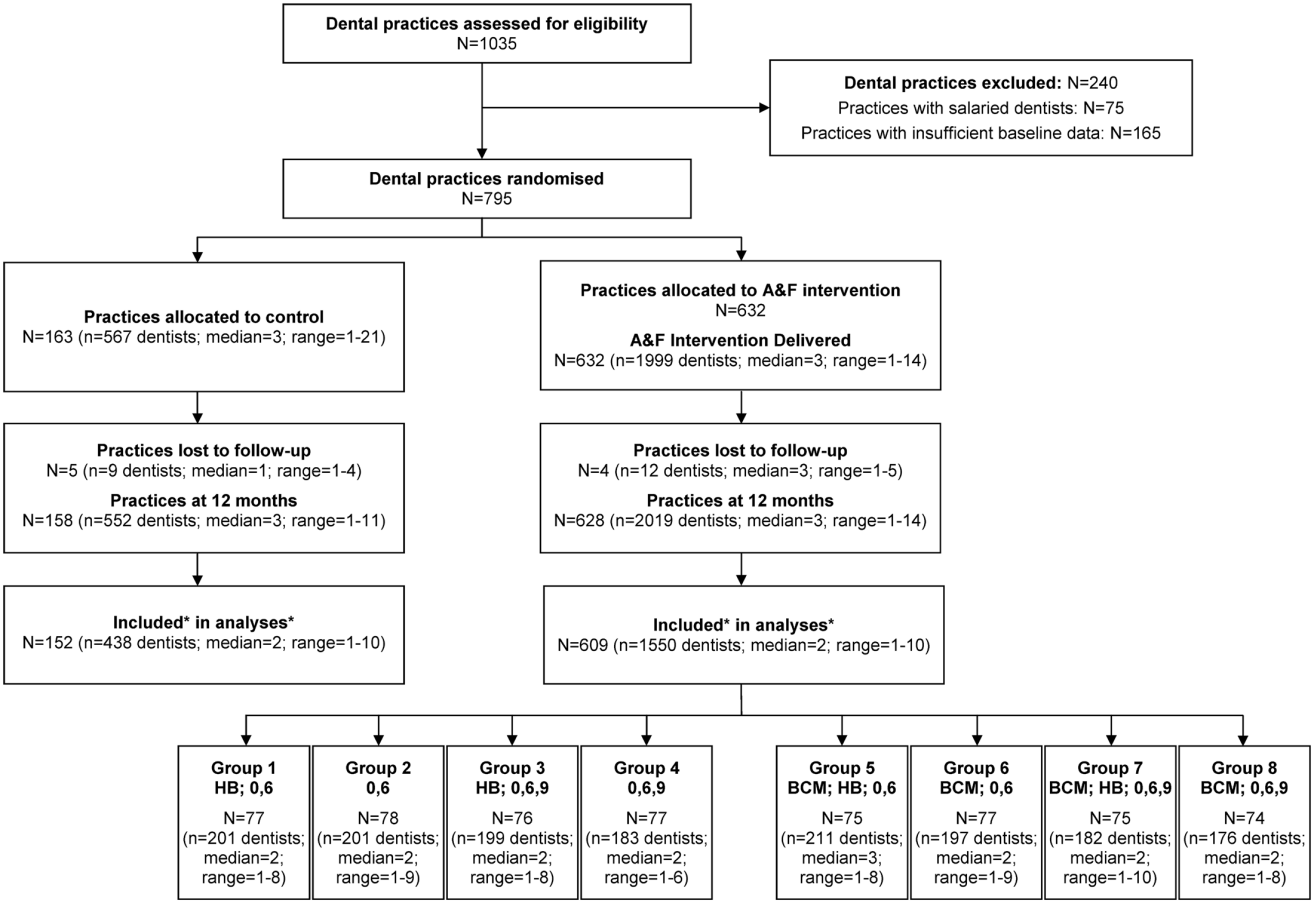
Practice and dentist flow diagram. Abbreviations: A&F—audit and feedback comprising a line graph plotting an individual dentist’s monthly antibiotic prescribing rate. BCM—written behaviour change message comprising text added below a dentist’s individualised line graph synthesising and reiterating national guidance recommendations for antibiotic prescribing. HB—health board comparator comprising addition of a line to the individualised line graph plotting the monthly antibiotic prescribing rate of all dentists in that dentist’s health board. 0,6—allocated intervention delivered at months 0 and 6. 0,6,9—allocated intervention delivered at months 0, 6, and 9. Trial Comparisons: Groups 1,2,3,4 versus Groups 5,6,7,8 test the written behaviour change message main effect. Groups 1,3,5,7 versus Groups 2,4,6,8 test the health board comparator main effect. Groups 1,2,5,6 versus Groups 3,4,7,8 test the frequency of feedback main effect. Dentists without both baseline and follow-up data were not included in the analyses (control *n* = 114; intervention *n* = 469). Practices without at least one dentist with both baseline and follow-up data were not included in the analysis (control *n* = 6; intervention *n* = 19).

All 1,035 locations providing dental services across Scotland were assessed for eligibility. Seventy-five practices with salaried dentists and 165 practices without a minimum of 6 mo of NHS treatment claim data in the 12 mo prior to the baseline intervention were excluded. A total of 795 practices (2,566 dentists) were randomised—632 (1,999 dentists) to the intervention group and 163 (567 dentists) to the control group. Average (median) cluster size in the intervention group and control group was 3 (intervention min = 1, max = 14; control min = 1, max = 21) (Fig 2).

All practices randomised to an intervention group received the initial feedback in May 2013. At 6 mo, the intervention was delivered to 629 practices (2,055 dentists), and at 9 mo, 316 practices (996 dentists) received the intervention. There was minimal loss to follow-up. Nine (1%) practices in total (intervention = 4; control = 5) closed during the 12 mo of the trial’s intervention delivery phase. In the remaining 786 practices (intervention = 628; control = 158), some dentists included at baseline had no follow-up data due to their leaving the practice, while other dentists joined trial practices during the trial and therefore had no baseline data. All dentists who had incomplete data (469 intervention dentists; 114 control dentists) were dropped from the analyses. In 19 intervention and 6 control practices, all dentists had incomplete data. This left 1,550 intervention dentists (609 practices) and 438 control dentists (152 practices) for inclusion in the analyses (Fig 2).

### Baseline

At baseline, NHS treatment claims (mean = 1,247) in the control group during the 12 months prior to delivery of the A&F intervention at month 0 (May 2012 to April 2013) were lower than in the intervention group (mean = 1,340). Prescribing rates were similar across the two groups (Table 1). Approximately 16% of the practices in each group were single-handed.

**Table 1:** Dentist level baseline information—control versus intervention.

	Control			Intervention (All)		
May 2012 to April 2013	*n*	Mean^1^	SD	*n*	Mean^1^	SD
Total number of claims	449	1,247.2	938.2	1,569	1,339.5	1,063.5
Total number of antibiotic items	507	133.3	116.8	1,903	140.9	132.0
Total defined daily doses of antibiotics	507	635.9	644.0	1,903	666.3	693.4
Total number of amoxicillin 3g items	507	1.3	5.2	1,903	1.5	7.1
Total defined daily doses of amoxicillin 3g	507	7.5	31.5	1,903	8.6	43.6
Total number of broad spectrum antibiotics^2^	507	3.8	24.4	1,903	1.1	7.0
Total defined daily doses of broad spectrum antibiotics^2^	507	22.5	182.8	1,903	4.4	32.6
**Primary Outcome**						
Total number of antibiotic items dispensed per 100 claims	438	8.3	7.2	1,550	8.5	9.5
**Secondary Outcomes**						
Total defined daily doses of antibiotics dispensed per 100 claims	438	39.5	40.4	1,550	40.7	55.5
Total number of amoxicillin 3g items dispensed per 100 claims	438	0.1	0.4	1,550	0.1	1.3
Total defined daily doses of amoxicillin 3g dispensed per 100 claims	438	0.4	2.2	1,550	0.5	7.2
Total number of broad spectrum antibiotics dispensed per 100 claims^2^	438	0.2	0.8	1,550	0.1	0.8
Total defined daily doses of broad spectrum antibiotics dispensed per 100 claims^2^	438	0.8	4.6	1,550	0.4	3.6

^1^ All prescribing averages are weighted by total claims at follow-up

^2^ Broad spectrum/4C antibiotics have been combined and include Clindamycin, Co-amoxiclav, Clarithromycin, Cefalexin, and Cefradine

### Follow-Up

At follow-up, the average number of NHS treatment claims by dentists in the control group remained constant (mean = 1247). For dentists in the intervention group, average claims were lower than at baseline (mean = 1295).

### Primary Outcome

The rate of antibiotic prescribing by dentists receiving an A&F intervention was reduced from 8.5 items per 100 NHS treatment claims at baseline to 7.5 items per 100 NHS treatment claims at follow-up. Dentists in the control group also reduced antibiotic prescribing from 8.3 items per 100 NHS treatment claims to 7.9 items per 100 NHS treatment claims, giving an overall adjusted effect size of 0.47 (95% CI 0.09 to 0.85) fewer antibiotic items per 100 NHS treatment claims. The primary analysis revealed this to be a significant change (*p* = 0.01), representing a 5.7% reduction (95% CI -1.1% to -10.2%) in the antibiotic prescribing rate in the intervention group relative to the control group (Table 2). The intra-cluster correlation coefficient was estimated at 0.193 (95% CI 0.15 to 0.24).

**Table 2:** Summary results of all item antibiotic prescribing rates (primary outcome).

Primary outcome	Baseline Mean		Follow-Up Mean		Difference in Rate (95% CI)	*P*-Value	% Reduction from Baseline^a^ (95% CI)
	**Control**	**Intervention**	**Control**	**Intervention**			
All antibiotic items/100 claims	8.3	8.5	7.9	7.5	-0.47 (-0.85, -0.09)	0.014	-5.7% (-10.2%, -1.1%)
***Intervention components***	**No BCM Intervention**	**BCM Intervention**	**No BCM Intervention**	**BCM Intervention**			
BCM intervention versus no BCM intervention	8.5	8.5	7.7	7.2	-0.51 (-0.86, -0.16)	0.005	-6.1% (-10.4%, -1.9%)
	**No HB Comparator**	**HB Comparator**	**No HB Comparator**	**HB Comparator**			
HB comparator versus no HB comparator	8.4	8.6	7.5	7.4	-0.36 (-0.72, 0.01)	0.057	-4.3% (-8.6%, 0.1%)
	**0,6,9 mo A&F**	**0,6 mo A&F**	**0,6,9 mo A&F**	**0,6 mo A&F**			
0,6 mo A&F versus 0,6,9 mo A&F	8.2	8.7	7.3	7.6	0.002 (-0.35, 0.35)	0.989	0.02% (-4.2%, 4.2%)

^a^ All percentages standardised using control group baseline mean prescribing rate (8.3)

BCM, written behaviour change message; HB, health board; A&F, audit and feedback

In the intervention subgroups, a significant 6.1% reduction in the prescribing rate was observed for those who received the graphical A&F plus the written behaviour change message compared to those who received the graphical A&F only (95% CI -10.4% to -1.9%; *p* = 0.01). There was no significant difference in the prescribing rate of dentists who were provided with a health board comparator as part of their graphical A&F compared to dentists who did not receive the comparator (-4.3%; 95% CI -8.6% to 0.1%; *p* = 0.06). There was also no significant difference in the prescribing rate between dentists who received A&F at 0 and 6 mo and those who received A&F at 0, 6, and 9 mo (0.02%; 95% CI -4.2% to 4.2%; *p* = 0.99) (Table 2). There was no statistically significant evidence of two-way interaction effects for (i) dentists who received a health board comparator and received A&F at 0, 6, and 9 mo (-3.0%; 95% CI -11.4% to 5.4%; *p* = 0.484); (ii) dentists who received the written behaviour change message and received A&F at 0, 6, and 9 mo (+1.0%; 95% CI -7.6% to 9.6%; *p* = 0.809); and (iii) dentists who received the written behaviour change message and received a health board comparator (+8.2%; 95% CI -0.4% to 16.9%; *p* = 0.061).

Subgroup analyses exploring the possibility of effect moderation by pre-intervention levels of prescribing were not statistically significant (interaction -0.74; 95% CI -1.78 to 0.29; *p* = 0.159). There was no significant difference in prescribing rates from baseline by high prescribers (> upper quartile) in the intervention group compared to high prescribers in the control group (-11.6%; 95% CI -23.5% to 0.2%; *p* = 0.06). There was also no significant difference in prescribing rates by low baseline prescribers (< lower quartile) in the intervention group compared to control (–2.7%; 95% CI -7.0% to 1.6%; *p* = 0.22). There was no indication that the variability of antibiotic prescribing rates was changed in the intervention period (Levene’s test, *p* = 0.414).

### Secondary Outcomes

Table 3 presents the results from the secondary outcomes analyses. The DDD rate reduced by -2.60 per 100 NHS treatment claims, representing a 6.6% reduction (95% CI -12.5% to -0.7%; *p* = 0.03) in the intervention group relative to the control group. No other statistically significant differences were observed for the remaining secondary outcomes (Table 3). For groups receiving the various components within the A&F intervention, there were no significant differences in the secondary outcomes (Table 3).

**Table 3:** Summary results for secondary outcome prescribing rates.

Secondary Outcome 1	Baseline Mean		Follow-Up Mean		Difference in Rate (95% CI)	*P-*Value	% Reduction from Baseline (95% CI)
	**Control**	**Intervention**	**Control**	**Intervention**			
DDD (all antibiotics)/100 claims^a^	39.5	40.7	39.7	37.3	-2.60 (-4.92, -0.28)	0.028	-6.6% (-12.5%, -0.7%)
***Intervention components***	**No BCM Intervention**	**BCM Intervention**	**No BCM Intervention**	**BCM Intervention**			
BCM intervention versus no BCM intervention	39.7	41.7	38	36.7	-2.25 (-4.23, -0.27)	0.026	-5.7% (-10.7%, -0.7%)
	**No HB Comparator**	**HB Comparator**	**No HB Comparator**	**HB Comparator**			
HB comparator versus no HB comparator	41.4	40	37.2	37.4	-1.66 (-3.73, 0.42)	0.117	-4.2% (-9.4%, 1.1%)
	**0,6,9 mo A&F**	**0,6 mo A&F**	**0,6,9 mo A&F**	**0,6 mo A&F**			
0,6 mo A&F versus 0,6,9 mo A&F	39.3	42	36.1	38.4	-0.41 (-2.40, 1.58)	0.689	-1.0% (-6.1%, 4.0%)
**Secondary Outcome 2**	**Control**	**Intervention**	**Control**	**Intervention**			
Amoxicillin 3g/100 claims^b^	0.1	0.1	0.1	0.1	-0.02 (-0.05, 0.01)	0.213	-26.0% (-64.9%, 13.0%)
***Intervention components***	**No BCM Intervention**	**BCM Intervention**	**No BCM Intervention**	**BCM Intervention**			
BCM intervention versus no BCM intervention	0.1	0.1	0.1	0.1	-0.01 (-0.03, 0.01)	0.460	-13.0% (-39.0%, 13.0%)
	**No HB comparator**	**HB comparator**	**No HB comparator**	**HB comparator**			
HB comparator versus no HB comparator	0.1	0.1	0.1	0.1	-0.01 (-0.03, 0.02)	0.670	-13.0% (-39.0%, 26.0%)
	**0,6,9 mo A&F**	**0,6 mo A&F**	**0,6,9 mo A&F**	**0,6 mo A&F**			
6 versus 3 monthly feedbacks	0.1	0.1	0.1	0.1	-0.01 (-0.03, 0.01)	0.437	-13.0% (-39.0%, 13%)
**Secondary Outcome 3**	**Control**	**Intervention**	**Control**	**Intervention**			
DDD (Amoxicillin 3g)/100 claims^c^	0.4	0.5	0.5	0.4	-0.14 (-0.35, 0.06)	0.179	-31.8% (-79.5%, 13.6%)
***Intervention components***	**No BCM Intervention**	**BCM Intervention**	**No BCM Intervention**	**BCM Intervention**			
BCM intervention versus no BCM intervention	0.5	0.6	0.3	0.4	-0.04 (-0.18, 0.11)	0.601	-9.1% (-40.9%, 25.0%)
	**No HB Comparator**	**HB Comparator**	**No HB Comparator**	**HB Comparator**			
HB comparator versus no HB comparator	0.6	0.5	0.4	0.3	-0.04 (-0.19, 0.10)	0.564	-9.1% (-43.2%, 22.7%)
	**0,6,9 mo A&F**	**0,6 mo A&F**	**0,6,9 mo A&F**	**0,6 mo A&F**			
0,6 mo A&F versus 0,6,9 mo A&F	0.4	0.6	0.3	0.4	-0.06 (-0.19, 0.08)	0.39	-13.6% (-43.2%, 18.2)
**Secondary Outcome 4**	**Control**	**Intervention**	**Control**	**Intervention**			
Broad spectrum antibiotics/100 claims^d^	0.2	0.1	0.1	0.1	-0.05 (-0.12, 0.03)	0.228	-33.3% (-80.0%, 20.0%)
***Intervention Components***	**No BCM Intervention**	**BCM Intervention**	**No BCM Intervention**	**BCM Intervention**			
BCM intervention versus no BCM intervention	0.1	0.1	0.1	0.1	-0.01 (-0.03, 0.02)	0.552	-6.7% (-20.0%, 13.3%)
	**No HB Comparator**	**HB Comparator**	**No HB Comparator**	**HB Comparator**			
HB comparator versus no HB comparator	0.1	0.1	0.1	0.1	0.003 (-0.02, 0.03)	0.81	2.0% (-13.3%, 20.0%)
	**0,6,9 mo A&F**	**0,6 mo A&F**	**0,6,9 mo A&F**	**0,6 mo A&F**			
0,6 mo A&F versus 0,6,9 mo A&F	0.1	0.1	0.1	0.1	-0.003 (-0.03, 0.02)	0.753	-2.0% (-20.0%, 13.3%)
**Secondary Outcome 5**	**Control**	**Intervention**	**Control**	**Intervention**			
DDD (Broad spectrum antibiotics)/100 claims^e^	0.8	0.4	0.8	0.3	-0.27 (-0.64, 0.10)	0.152	-33.3% (-79.0%, 12.3%)
***Intervention Components***	**No BCM Intervention**	**BCM Intervention**	**No BCM Intervention**	**BCM Intervention**			
BCM intervention versus no BCM intervention	0.4	0.3	0.3	0.2	-0.05 (-0.17, 0.07)	0.405	-6.2% (-21.0%, 8.6%)
	**No HB Comparator**	**HB Comparator**	**No HB Comparator**	**HB Comparator**			
HB comparator versus no HB comparator	0.3	0.4	0.3	0.2	0.04 (-0.09, 0.16)	0.565	4.9% (-11.1%, 19.8%)
	**0,6,9 mo A&F**	**0,6 mo A&F**	**0,6,9 mo A&F**	**0, 6 mo A&F**			
0,6 mo A&F versus 0,6,9 mo A&F	0.2	0.4	0.2	0.3	-0.02 (-0.13, 0.08)	0.660	-2.5% (-16.0%, 9.9%)

^a^ Percentages standardised using control group mean prescribing rate (39.5);

^b^ standardised using 0.077;

^c^ standardised using 0.44;

^d^ standardised using 0.15;

^e^ standardised using 0.81

DDD, defined daily dose; BCM, written behaviour change message; HB, health board; A&F, audit and feedback

### Process Evaluation

The findings from the process evaluation are reported in S1 Table. In summary, dentists reacted positively to receiving the A&F intervention, although views varied according to the type of intervention received. Participants believed that the graph was easy to understand and the feedback was useful and beneficial to manage their prescribing behaviour. The findings support the results from the statistical analyses in relation to the inclusion of guidance or instruction as given in the written behaviour change intervention. Dentists also expressed a preference for a comparator to be included in their feedback but expressed no strong preference for receiving feedback more than twice a year.

As a direct result of receiving the A&F intervention, some dentists had initiated discussions with colleagues to review and agree on prescribing patterns, while others had made decisions to delay treatment with antibiotics. Most participants believed the intervention had changed their antibiotic prescribing practices. No dentist who participated in the process evaluation reported any unintended consequences or harm as a result of receiving their A&F. Suggestions such as a more localised or additional comparators and the inclusion of patient data were proposed as ways in which the intervention could be modified or improved.

## Discussion

This study assessed the effectiveness of individualised A&F interventions on the translation into practice of guidance recommendations on antibiotic prescribing in dental primary care across an entire healthcare system (Scotland). For dentists in practices exposed to an A&F intervention, significant reductions in the total number of antibiotics per 100 NHS treatment claims and the DDD prescribing rates were observed, relative to dentists in control group practices. If the benefit was extrapolated to all dentists in Scotland, this would equate to approximately 20,000 fewer antibiotic items over a 12-mo period. Subgroup analyses found that the A&F intervention that demonstrated the greatest impact was provision of a line graph depicting a dentist’s monthly antibiotic prescribing rate plus a written behaviour change message that synthesised and reiterated national guidance recommendations for dental antibiotic prescribing. There was no statistically significant difference between dentists who received a line graph of their own antibiotic prescribing rate compared to the prescribing rate of all dentists in their health board with those who were not shown the comparator. However, some dentists who participated in the process evaluation expressed a preference for inclusion of a health board comparator, and, therefore, this could also be considered. There was no evidence to suggest that feedback provided more frequently than every 6 mo had any additional impact on antibiotic prescribing rates.

The effects of the intervention varied across the individual secondary outcomes being tested. There was a statistically significant reduction in the DDD prescribing rate of dentists who received an A&F intervention compared to control group dentists. However, the antibiotic item prescribing rates and DDD prescribing rates of amoxicillin 3g and the broad spectrum antibiotics did not differ significantly between the intervention and control groups. There was also no effect moderation by pre-intervention levels of prescribing, though any moderation may be contaminated by regression to the mean effects.

We previously discussed the strengths and limitations of this study design in the published protocol [15]. These included the study’s innovation in its use of routinely collected electronic healthcare data across all stages of the trial design. In particular, administrative datasets (MIDAS and PRISMS) were used to identify the study population, apply eligibility criteria, carry out stratified randomisation, generate individualised feedback for the trial intervention, and analyse trial outcomes. Methodological strengths of this design include minimisation of assessment reactivity (e.g., non-contact recruitment of trial participants, no-contact (postal) delivery of the trial intervention, no self-report measures, and no opportunity for researchers to influence the antibiotic prescribing rates presented in the feedback [26]. These features reduce the potential for pre- and post-randomisation sources of bias associated with recruitment, baseline assessment activities, exposure to study conditions, and assessment at follow-up.

Another previously discussed [15] methodological strength of the RAPiD trial is that it operationalised published recommendations for the design of A&F intervention studies [27]. Specifically, RAPiD adopted “best practices” for A&F components (e.g., data are individualised, based on recent performance, and new data are presented over time), applied relevant theory to the development of the written behaviour change intervention [27], and investigated further optimisation interventions in conjunction with a concurrent process evaluation [28]. The addition of the qualitative interviews also contributed to the interpretation of the trial results and a better understanding of how the intervention works. Process evaluation interviews were conducted with 30 purposively sampled dentists from both intervention and control practices. Most were positive about receiving the intervention and found it useful and easy to understand. Many reported positive changes in antibiotic prescribing behaviour, both at the individual and practice level.

While numerous studies [29–34] and reviews [35–37] have evaluated interventions to enhance prescribing practices, to our knowledge none have evaluated the effects of feedback A&F interventions on antibiotic prescribing rates in this way and at scale in primary care. All 795 eligible general dental practices across all health boards in Scotland were included in the trial. With minimal loss to follow-up, we can be confident about the generalisability of the results.

One potential limitation is the relatively short duration of the trial. Although there is currently no funding to enable continued delivery of antibiotic prescribing A&F to intervention group dentists, the use of routinely collected datasets allows evaluation of the sustainability of the results over a longer period of time. In order to do this, continued access to PRISMS and MIDAS data has been secured until April 2017, enabling follow-up in the longer term.

The use of routinely collected datasets presents limitations as well as strengths. For example, as discussed in the published protocol [15], PRISMS collects dispensing rather than prescribing data, and MIDAS is a repository for remuneration data rather than treatment provided. Claims for payment for dental treatment are submitted at the end of a course of treatment. In some instances, a course of treatment may be delivered over a number of weeks, while an antibiotic may be prescribed and dispensed at any time during this period. Thus, only a proxy measure of the monthly rate of antibiotic prescribing could be obtained from these datasets.

Importantly, the data used in this study allowed us only to consider effects on the total number of antibiotic items prescribed. The data did not provide any information about the oral health status or presenting condition of the patient. Therefore, although some intervention group dentists did report positive changes in antibiotic prescribing behaviour, it is possible that the interventions had no impact or a negative impact on the quality or appropriateness of dentists’ antibiotic prescribing. Indeed, although the process evaluation did not identify any unintended consequences or harms, no other exploration of this possibility was carried out, and some unintended consequences or harms may not have been identified.

The results are important in the context of the global health concern around antibiotic resistance and recent steady increases in antibiotic prescribing by healthcare professionals. Studies [29,38] in other healthcare settings have demonstrated that reductions in antibiotic prescribing rates can lead to associated reductions in antimicrobial resistance. The RAPiD trial is a relatively straightforward, low-cost public health and patient safety intervention that could help the entire healthcare profession address the increasing challenge of antimicrobial resistance.

This study has successfully demonstrated the potential to fully embed RAPiD-style A&F within routine service delivery. Through its collaborative links with dental healthcare policymakers, TRiaDS is currently identifying the best way to take this forward. This will provide a mechanism to test and evaluate a range of interventions to further improve dentists’ antibiotic prescribing.

The findings also support the emerging view [39] that multifaceted interventions might not be more effective than single interventions (as used in our study). The overall reduction in antibiotic prescribing observed in the intervention group compared to the control group is similar in magnitude to that found in multifaceted interventions that use audit and feedback in their design [29]. It is likely that multifaceted interventions are more complex and costly to deliver and maintain than the approach adopted in this study.

In conclusion, the rigorous trial design and the theory-based qualitative process evaluation provide a robust evaluation of A&F in antibiotic prescribing in dental primary care. It has helped elucidate the mechanisms by which A&F works best and has created a platform for further research to adapt and refine the intervention to achieve maximum benefit. This study paves the way for applying the methodology in different contexts with different target behaviours, not only in dentistry but in other healthcare settings as well.

## Supporting Information

S1 Fig Example audit and feedback chart, including behaviour change message and health board comparator. (PDF)

S1 Table Summary of qualitative data from process evaluation. (PDF)

S1 Text Development of the TRiaDS written behaviour change intervention. (PDF)

S2 Text CONSORT checklist. (PDF)

S3 Text TIDieR checklist. (PDF)

## Abbreviations

A&F: audit and feedback
BCT: behaviour change technique
DDD: defined daily dose
GAfREC: Governance Arrangements for Research Ethics Committees
MIDAS: Management Information and Dental Accounting System
NHS: National Health Service
PRISMS: Prescribing Information System for Scotland
RAPiD: Reducing Antibiotic Prescribing in Dentistry
SDCEP: Scottish Dental Clinical Effectiveness Programme
TRiaDS: Translation Research in a Dental Setting
