# Supplementary material for: An Audit and Feedback Intervention for Reducing Antibiotic Prescribing in General Dental Practice: The RAPiD Cluster Randomised Controlled Trial
Source: PLoS Med. 2016 Aug 30;13(8):e1002115. doi: 10.1371/journal.pmed.1002115 (PMC5004857; doi:10.1371/journal.pmed.1002115)
Supplement: S1 Fig — (PDF) [file pmed.1002115.s001.pdf]

S1 Fig. Example Audit and Feedback Chart Including Behaviour Change Message and Health Board Comparator

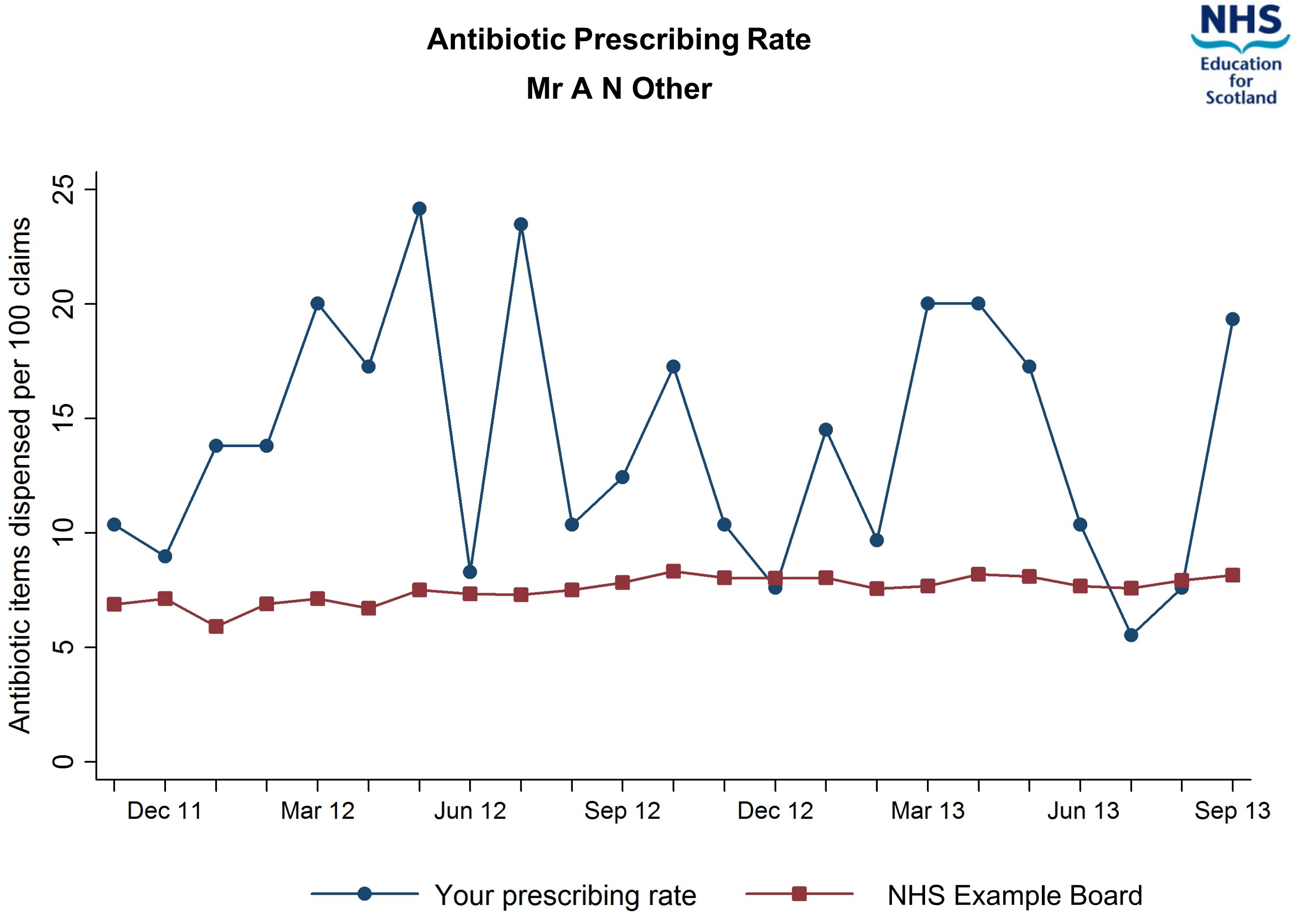

Your prescribing rate is your monthly number of antibiotic items dispensed multiplied by 100 and divided by the average monthly number of claims made on your ordinary lists at this practice between November 2011 and September 2013. The health board rate is the overall ordinary list prescribing rate for current dentists in non-salaried practices in NHS Example Board. (Source: ISD Scotland. Data as at January 2014)

Prescribing courses of antibiotic treatment can encourage the development of antimicrobial resistance and therefore must be kept to a minimum.

As a first step in the treatment of bacterial infections, use local measures. For example, drain pus if present in dental abscesses by extraction of the tooth or through root canals, and attempt to drain any soft-tissue pus by incision.

This should be the first step even if patients request antibiotics and even when time is short.

Antibiotics are appropriate for oral infections where there is evidence of spreading infection, systemic involvement or persistent swelling despite local treatment.

Use antibiotics in conjunction with, and not as an alternative to, local measures.

If you would like to discuss any part of this feedback please contact: (Contact details provided were the contact's name, telephone and email).
