# Supplementary material for: An Audit and Feedback Intervention for Reducing Antibiotic Prescribing in General Dental Practice: The RAPiD Cluster Randomised Controlled Trial
Source: PLoS Med. 2016 Aug 30;13(8):e1002115. doi: 10.1371/journal.pmed.1002115 (PMC5004857; doi:10.1371/journal.pmed.1002115)
Supplement: S1 Table — (PDF) [file pmed.1002115.s002.pdf]

## S1 Table. Summary of qualitative data from process evaluation

| Themes | Summary of responses | Sample Quote (with participant number) |
|---|---|---|
| General views on A&F[1] | ▪ Useful and easy to understand<br>▪ Useful indicator for managing own prescribing practise (i.e. increase or decrease if needed)<br>▪ Nice to compare own data with that of other dentists/colleagues within or out with the practice | *It's [A&F] useful, well, in fact it's very useful, I would say, because it does indicate on a graphic form or a chart, you can see whether or not you're consistent or you're over-prescribing or under-prescribing or anything else and can compare it with other colleagues as well. It's, I mean, the first time that this sort of information has been available, you know, for a while, so it does, it is quite a useful thing to get (13474)*<br><br>*It [A&F] was really nice. I see how much we are doing and how much in general dentists are doing; other dentists are doing in total. (10117)*<br><br>*I think it [A&F] was useful because it gives you an idea about how you are doing in a year, and it also draw a signal if you prescribe too much, so I think it's useful. (13388)* |
| Validity &/or reliability of data | ▪ It reassured their own prescribing figures consistent with those in the feedback<br>▪ It was not clear how their prescribing level was showing higher than expected because e.g. don't claim for all patients; treating a large number of private patients or children | *I record the number of prescriptions that I issue per month. We have a sheet in the surgery here and the numbers were similar to what you had recorded or they probably should be exactly the same as what you'd recorded… (13477)*<br><br>*I had to contact where it came from to get details of the Y axis, 'cause I didn't fully understand it…The graph was very understandable, I just wasn't sure how they got the data…They emailed me a couple of times, so it's clear now… (10868)* |
| The TRiaDS written behaviour change intervention was a useful refresher | ▪ It was very useful<br>▪ It was a refresher<br>▪ No new information was given | *Yes, aye, it [written behaviour change intervention] was useful. I mean it wasn't as useful as the graph, the graph was the main thing (11156)*<br><br>*It [written behaviour change intervention] was a wee bit of a refresher so I think it was good (10469)*<br><br>*I think there [written behaviour change intervention] was something about, giving an idea of what would be a good idea to do…I don't think the advice is anything new…I thought it had a tendency towards naivety…I don't think it was either useful ... (10841)* |

1 – Audit and Feedback

| Themes | Summary of responses | Sample Quote (with participant number) |
|---|---|---|
| A&F[1] can change prescribing practice | ▪ It keeps every dentist in check (i.e. how they are treating bacterial infections)<br><br>▪ It makes you think about other ways of treating patients rather than writing prescription<br><br>▪ It has resulted in personal decision to delay treatment with antibiotics and review patients instead<br><br>▪ It won't change behaviour as prescriptions are only written when its necessary | *I mean, I have seen antibiotics being prescribed for nothing at all and I'm not happy in my own practice to be done but unfortunately it takes a little bit of time to assess somebody else. So this sort of feedback will keep everybody in check (10116)*<br><br>*… sometimes if you're very under pressure, you've only got five or ten minutes to do something, you know, sometimes it is easier to write a prescription, but it [A&F] does stop and make you think, and see if you can do something else other than write a prescription. And that's really only because I've looked at my antibiotic prescribing levels in the A&F document (13474)*<br><br>*Well, mine went down significantly, so I actually did reduce the amount I gave … suppose you've got drainage, I would maybe give some antibiotics for fast resolution, and now I tend to see them back three days later to see how the swelling's gone down. But before I gave them antibiotics, …So I've decided to delay treatment with antibiotics, and get them back and see how they're doing (10841)*<br><br>*It's (A&F) not going to change my habits, terribly…I use prescriptions when I think they're necessary. I'm not likely to change that drastically (11355)* |
| Communicating & acting on goals | ▪ Never discussed with others and/or never set any goals<br><br>▪ Discussions with colleagues within/outwith practice | *I'm a sole practitioner actually and, to be honest, speaking to friends it didn't ever come up in conversation at all. So there wasn't any actually any formal or informal discussion with any colleagues at all (10968)*<br><br>*I didn't make any goals because according to those two lines mine was quite low so I'm going fine so I won't reduce… I would agree to the fact that if somebody was prescribing more than average then I would certainly think about reducing it (10117)*<br><br>*Yeah, we did discuss the A&F between me and three other dentists and the practice manager. We didn't set any goals as such in the way of figures but we did aim overall to reduce the incidences in which we were prescribing for patients with infection and always trying to deal with the infection using first-line methods (10469)* |

1 – Audit and Feedback

| Themes | Summary of responses | Sample Quote (with participant number) |
|---|---|---|
| Communicating & acting on goals (cont'd) | ▪ Discussions with practice colleagues to agree goals and review prescribing patterns<br><br>▪ Made personal goal to justify every prescriptions, stop any unnecessary prescribing or prescribe less | *We had a practice meeting in the surgery and we discussed this [A&F] and set goals, which would be first of all how many prescriptions we made per day and also the reason for prescribing it (10814)*<br><br>*I had set goals to view every prescription I write. To see whether or not the prescriptions for antibiotics I write were justifiable or whether there was some other treatment that I could do that would avoid writing a prescription for antibiotics.* |
| Recommendations for future A&F[1] studies | ▪ Providing A&F in a non-critical way to avoid resistance<br><br>▪ Providing guidance on how to minimise prescribing antibiotics<br><br>▪ Adding a comparator (i.e. National/local/other practitioners) would be useful to compare their prescribing level<br><br>▪ Assigning patient data along with A&F would allow for greater reflection on whether prescribing appropriately<br><br>▪ Breaking down of data by the number of prescriptions given (i.e. amoxicillin and metronidazole in one prescription) rather than the number of items/antibiotics prescribed because it might affect the result/graph<br><br>▪ Frequency of receiving A&F in the future: biannual to annual | *I think it [A&F] has to be done in a collegial way, rather than a critical way, so that when things are coming in; they're trying to be helpful. In other words, the carrot rather than the stick…Because if people feel it's a criticism they're either going to resist it or just go in the huff (10841)*<br><br>*If you're going to provide feedback you need to provide some sort of guidance in relation to how to minimise your prescribing rather than just giving you the information you need to provide ways to either educate or change your practice in order to minimise your prescribing (10600)*<br><br>*I would like to know more about other regions and other surrounding local dentists … not just what the whole lot [whole UK/Scotland] is doing… If there is an average for all the people around Scotland or England it doesn't give you much insight (10117)*<br><br>*It would have been better if you could have got the patients' list, then at least even as a sort of an audit project, you could actually look and see under what circumstances were people actually prescribed antibiotics and see if there was a pattern in that…(11542)*<br><br>*I think it would have been more sensible if it [A&F] had graphed the number of antibiotic prescriptions, not items. Because it magnifies it up when you're giving people, say for instance amoxicillin and flagyl, amoxicillin and metronidazole together, then that'll score two, whereas if it's in the one prescription and been given to the one patient at the same time, then I think that that should only score one (10841)*<br><br>*I suppose anything [frequency of sending A&F] from six months to a year, really. I suppose if we're doing it for the purposes of audit, you'd want something more frequently than every year, just, you know, just to see that you haven't lapsed into the bad old ways, or bad habits (13308)* |

1 – Audit and Feedback
